# Basic Residues R260 and K357 Affect the Conformational Dynamics of the Major Facilitator Superfamily Multidrug Transporter LmrP

**DOI:** 10.1371/journal.pone.0038715

**Published:** 2012-06-20

**Authors:** Wei Wang, Hendrik W. van Veen

**Affiliations:** Department of Pharmacology, University of Cambridge, Cambridge, United Kingdom; Russian Academy of Sciences, Institute for Biological Instrumentation, Russian Federation

## Abstract

Secondary-active multidrug transporters can confer resistance on cells to pharmaceuticals by mediating their extrusion away from intracellular targets via substrate/H^+^(Na^+^) antiport. While the interactions of catalytic carboxylates in these transporters with coupling ions and substrates (drugs) have been studied in some detail, the functional importance of basic residues has received much less attention. The only two basic residues R260 and K357 in transmembrane helices in the Major Facilitator Superfamily transporter LmrP from *Lactococcus lactis* are present on the outer surface of the protein, where they are exposed to the phospholipid head group region of the outer leaflet (R260) and inner leaflet (K357) of the cytoplasmic membrane. Although our observations on the proton-motive force dependence and kinetics of substrate transport, and substrate-dependent proton transport demonstrate that K357A and R260A mutants are affected in ethidium-proton and benzalkonium-proton antiport compared to wildtype LmrP, our findings suggest that R260 and K357 are not directly involved in the binding of substrates or the translocation of protons. Secondary-active multidrug transporters are thought to operate by a mechanism in which binding sites for substrates are alternately exposed to each face of the membrane. Disulfide crosslinking experiments were performed with a double cysteine mutant of LmrP that reports the substrate-stimulated transition from the outward-facing state to the inward-facing state with high substrate-binding affinity. In the experiments, the R260A and K357A mutations were found to influence the dynamics of these major protein conformations in the transport cycle, potentially by removing the interactions of R260 and K357 with phospholipids and/or other residues in LmrP. The R260A and K357A mutations therefore modify the maximum rate at which the transport cycle can operate and, as the transitions between conformational states are differently affected by components of the proton-motive force, the mutations also influence the energetics of transport.

## Introduction

Multidrug resistance originating from active efflux of pharmaceuticals from cells is a major obstacle in the treatment of microbial infections and human cancers [1]–[4]. Active drug extrusion is mediated by multidrug transporters, which recognize a wide variety of structurally unrelated compounds [5]–[7]. On the basis of bioenergetic and structural criteria, multidrug transporters can be divided into two major classes: (i) primary-active ABC transporters, which utilize the free energy of ATP binding/hydrolysis to efflux toxic substrate, and (ii) secondary-active transporters, which mediate drug extrusion in a coupled exchange with H^+^ and/or Na^+^ ions [8]. There are many questions regarding the mechanism by which these transport systems operate.

The multidrug transporter LmrP from *Lactococcus lactis* is a secondary-active transporter that is a member of the widely occurring Major Facilitator Superfamily (MFS) [9]. LmrP is a 408-amino-acid polypeptide that is predicted to contain 12 transmembrane helices (TMHs). Its drug efflux activity was first found in genetic screens based on its ability to confer resistance on *Escherichia coli* to high concentrations of a monovalent amphiphilic ethidium cation [10]. The transporter can utilise both the membrane potential (Δψ, interior negative) and the chemical proton gradient (ΔpH, interior alkaline) of the proton-motive force (Δp) to mediate the efflux of amphiphilic substrates from cells. The transport mechanism was inferred to be electrogenic antiport, whereby monovalent cationic ethidium^+^ and tetraphenylphosphonium (TPP^+^) move in exchange for two or more protons [11]. In the absence of the Δp, LmrP can also mediate facilitated transport of substrates down their chemical gradient [12], [13]. During active and facilitated transport, LmrP is thought to mediate the transbilayer movement of substrates via a rigid body motion of the N- and C-terminal halves with a concomitant alternating access of substrate-binding sites to the inside surface and outside surface of the cytoplasmic membrane, in an analogous fashion as proposed for LacY, LeuT and other secondary-active transporters [14]–[17].

Studies on drug/proton antiporters including EmrE, MdfA, and QacA have highlighted the importance of carboxyl residues in TMHs in interactions with cationic substrates and/or protons [18]–[20]. Previously a 3-D homology model of LmrP with high geometrical quality was constructed [12], based on the high-resolution, inward-facing structure of the glycerol-3P/Pi antiporter GlpT from *Escherichia coli* [21]. GlpT and LmrP are evolutionary related in an unambiguous manner, and the modeling suggested that the proteins have a comparable protein fold. Similar to the GlpT template, inward-facing LmrP is predicted to expose an internal cavity between its N- and C-terminal halves to the inner leaflet of the cytoplasmic membrane. The carboxylates exposed on the surface of this cavity in LmrP are organized into two distinct clusters containing additional neutral-polar and aromatic residues (see Fig. 2 in [12]). Cluster 1 in the C-terminal half contains D235 and E327 in immediate proximity of each other, and is located near the apex of the cavity, while Cluster 2 in the N-terminal half contains D142. Functional analyses of mutants suggest that D142 acts a dedicated proton-binding site, whereas D235 and E327 are part of a flexible multidrug binding surface that can interact with cationic substrates and protons [22].

**Figure 1:**
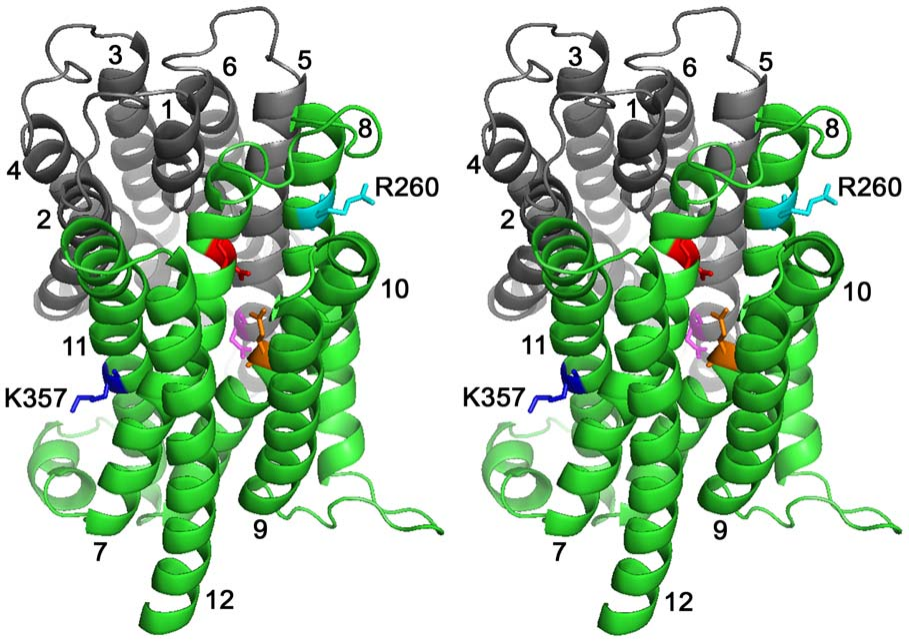
Stereoview of LmrP showing positions of basic residues R260 and K357. Ribbon representation of inward-facing LmrP [12] viewed sideways from the external side of the membrane. Transmembrane helices (TMHs) 1 to 6 in the N-terminal half (in grey) and TMHs 7 to 12 in the C-terminal half (in green) are numbered. R260 (in light blue, TMH 8) and K357 (in dark blue, TMH 11) are labelled, and are shown in stick representations. The three catalytic carboxylates D142 (TMH 5), D235 (TMH 7) and E327 (TMH 10) are coloured magenta, red, and orange, respectively.

In contrast to the more extensively characterized roles of carboxylates in secondary-active multidrug transporters, few studies have focused on the role of basic residues in these systems. LmrP is predicted to contain only two basic residues in TMHs, R260 (TMH 8) and K357 (TMH 11) in the C-terminal half (Fig. 1). Different from the catalytic carboxylates (D142, D235 and E327) that are present on the surface of the interior chamber in our inward-facing model, K357 and R260 are located on the outer surface near the phospholipid head group region of the inner leaflet (K357) and outer leaflet (R260) of the cytoplasmic membrane. Here, we characterized the functional role of K357 and R260 in multidrug transport by LmrP.

## Results

### Expression of LmrP Mutants

**Figure 2:**
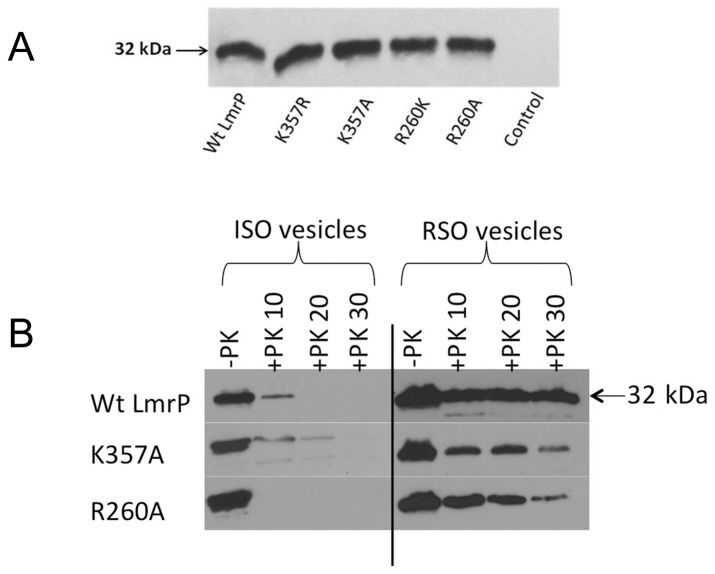
Expression and topology of mutant LmrP proteins. **A**, Western blot of total membrane proteins in inside-out membrane vesicles (ISOVs) (15 µg of protein/lane) probed with anti-His_5_-tag antibody, showing equal expression of Wt and mutant LmrP proteins in the plasma membrane of *L. lactis* NZ9000 Δ*lmrA* Δ*lmrCD* (with an apparent molecular mass on 10% SDS-PAGE of about 32 kDa), and lack of this expression in controls cells. **B,** The availability of the C-terminal His-tag in Wt LmrP, and the K357A and R260A mutants to cleavage by proteinase K (*+ PK*) at the external side of ISOVs and right-side-out membrane vesicles (RSOVs) was tested during incubations of 10, 20 and 30 min. Subsequently, remaining C-terminal His-tag was detected by Western blotting. Control incubation (lane 1, *-PK*) is without proteinase K. Equal loading of the lanes was confirmed by densitometry of the same samples on Coomassie-stained SDS-PAGE (not shown).

To study the functional role of K357 and R260 in the C-terminal half of LmrP, we generated single mutant proteins in which these residues were each replaced by a nonpolar A. In addition, K357 and R260 were substituted in a charge-conservative fashion (Table 1). Wildtype (Wt) LmrP and K357A, K357R, R260A, and R260K mutant proteins were expressed in *L. lactis* strain NZ9000 Δ*lmrA* Δ*lmrCD* [23] under the control of a nisin A-inducible promoter in the expression plasmid pNZ8048 [24]. Coomassie-stained SDS-PAGE and western blot analysis of equal amounts of total membrane proteins in inside-out membrane vesicles (ISOVs) demonstrated that the expression level of the mutant proteins in the cytoplasmic membrane was similar to that of Wt (Fig. 2A). As the presence of basic residues in TMHs of membrane proteins can act as potent determinants of membrane topology [25], [26], we compared the topology of the C-terminal half of Wt and mutant LmrP proteins through studies on the accessibility of the intracellular C-terminal His_6_-tag to digestion with proteinase K in preparations of well-defined ISOVs and right-side-out membrane vesicles (RSOVs). Similar to Wt LmrP, complete proteolytic removal of the His-tag from the mutant LmrP proteins was achieved in ISOVs, but not in RSOVs (Fig. 2B). These data demonstrate the intracellular location of the C-terminus for all proteins. Together with the observed functionality of the mutants in Δp-dependent and facilitated transport modes (see below), these results suggest that the topology of LmrP remains intact following the substitutions of K357 and R260.

**Table 1:** Primers used for site-directed mutagenesis.

No	Primer	Sequence 5′-3′
1	C270S Fw	C ATA TAT TTG ATT TTA GCT **AGC** GTA TTA GTT GTC
2	C270S Re	GAC A AC TAA TAC **GCT** AGC TAA AAT CAA ATA TAT G
3	I34C Fw	CT TCA ATG ACC **TGT** TAT TAT AAT C
4	I34C Re	G ATT ATA ATA **ACA** GGT CAT TGA AG
5	V240C Fw	GAT AAT TTT TTA CCT **TGC** CAT TTA TCG AAT AG
6	V240C Re	CT ATT CGA TAA ATG **GCA** AGG TAA AAA ATT ATC
7	K357R Fw	GCA ATT **AGA** ATG CCA ATT GCA TCA ATT C
8	K357R Re	TGG CAT **TCT** AAT TGC TGC AAC GCC ATT A
9	K357A Fw	GGC GGT GCA GCA ATT **GCA** ATG CCA ATT
10	K357A Re	AAT TGG CAT **TGC** AAT TGC TGC AAC GCC
11	R260K Fw	T GGA CAA **AAG** ATG CTC ACC ATA TAT TTG
12	R260K Re	AG CAT **CTT** TTG TCC ATA GAT TTC AAA AA
13	R260A Fw	GAA ATC TAT GGA CAA **GCG** ATG CTC ACC
14	R260A Re	GGT GAG CAT **CGC** TTG TCC ATA GAT TTC

### Mutations Affect Ethidium Transport

**Figure 3:**
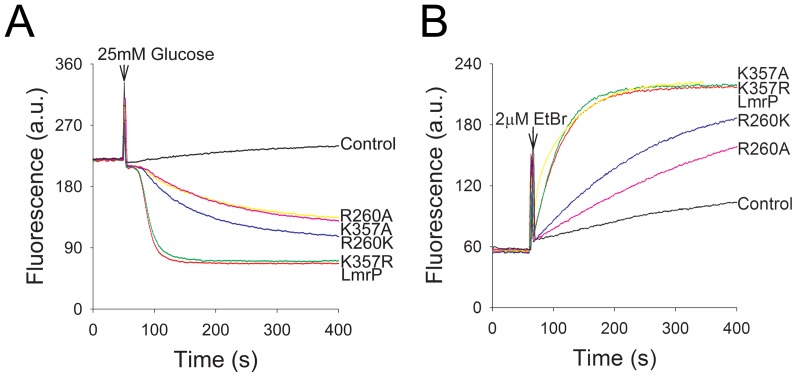
Ethidium transport in intact cells. **A,** ATP-depleted cells expressing Wt LmrP, the mutants K357R, K357A, R260K, or R260A, or no LmrP protein (control) were preloaded with 2 µM ethidium in 50 mM KP_i_ buffer (pH 7.0) containing 5 mM MgSO_4_. Glucose (25 mM) was added at the time point indicated, to allow the generation of metabolic energy in the cells. The initial rates of active efflux were calculated over the first 20 s during which transport was linear. **B,** Facilitated ethidium influx in ATP-depleted cells. Ethidium bromide (2 µM) was added at the time point indicated. The initial influx rates were calculated over the first 40 s of linear uptake with the exception of K357A for which the rate was determined over the first 20 s. Data in the main text represent the mean ± SEM of three independent experiments (n = 3). Ethidium transport was measured by fluorimetry.

Ethidium is a permanent monovalent cation, and a known substrate of LmrP. The dye undergoes a fluorescence intensity enhancement upon its intercalation in intracellular nucleic acids. The effects of K357 and R260 replacements on LmrP-mediated ethidium transport were first monitored in intact cells. When ATP-depleted *L. lactis* Δ*lmrA* Δ*lmrCD* cells, preloaded with 2 µM ethidium, were incubated with 25 mM glucose, a Δp (interior alkaline and negative) was generated by the proton pump activity of the F_1_F_O_-H^+^ ATPase. The LmrP proteins couple this Δp to ethidium extrusion from the cell. The efflux activity for K357R mutant (2.51±0.43 a.u./s) was similar to that of Wt LmrP (2.58±0.63 a.u./s). Although reduced compared to Wt LmrP, the R260K mutant (0.39±0.14 a.u./s), R260A mutant (0.22±0.03 a.u./s) and K357A mutant (0.28±0.08 a.u./s) exhibited a substantial rate of ethidium efflux compared to control (Fig. 3A).

To study downhill, facilitated transport of substrates by LmrP in the absence of Δp, the facilitated influx of ethidium in intact cells was measured at an external ethidium concentration of 2 µM. The influx rates for K357R mutant (1.92±0.12 a.u./s) and K357A mutant (1.98±0.12 a.u./s) were similar to Wt LmrP (1.78±0.30 a.u./s) (Fig. 3B). The influx rates for the R260K mutant (0.31±0.05 a.u./s) and R260A mutant (0.14±0.02 a.u./s) were decreased compared to that of Wt LmrP (P<0.05) (Fig. 3B). However, all mutants mediated significantly more ethidium influx than control (0.04±0.02 a.u./s) (P<0.05) (Fig. 3B).

**Figure 4:**
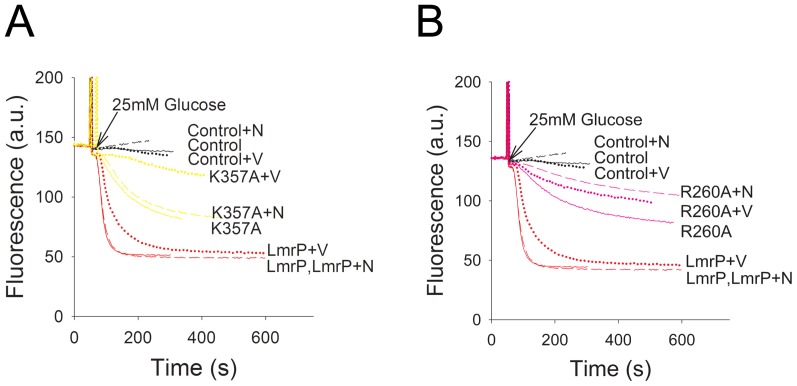
Dependence of active ethidium efflux on the composition of the proton-motive force (Δp). Ethidium transport was measured as described in the legend to Fig. 3A for cells without LmrP (control) or with Wt LmrP, or expressing mutant K357A (panel **A**) or R260A (panel **B**). Composition of Δp (interior negative and alkaline) in cells in KPi buffer was manipulated by the addition of 1 µM of the ionophores nigericin (+N) or valinomycin (+V) that dissipate the ΔpH and Δψ, respectively. Traces represent observations in at least four independent experiments (n = 4).

To further analyse the functional role of K357 and R260, we examined the ability of the mutant proteins to export ethidium in the presence of single components of the Δp, i.e. either the ΔpH or the Δψ, through the addition of ionophores valinomycin or nigericin (1 µM each) [27]. Valinomycin is a mobile carrier in the membrane that catalyses the electrogenic movement of K^+^ and dissipates the Δψ when cells are suspended in high K^+^ (50 mM) buffer. In contrast, nigericin selectively dissipates the ΔpH under these conditions by catalyzing electroneutral K^+^/H^+^ exchange across the membrane. Our studies revealed that dissipation of either component of the Δp did not significantly affect Wt LmrP-mediated transport from cells preloaded with 2 µM ethidium (Fig. 4A,B). In the case of the K357A mutant, dissipation of the ΔpH by nigericin had a small effect on ethidium efflux whereas valinomycin significantly attenuated transport activity (Fig. 4C). This finding indicates that the K357A mutation induces a shift to Δψ dependence over ΔpH dependence compared to Wt LmrP. Finally, the addition of nigericin gave the most significant reduction in ethidium efflux by R260A LmrP compared to the addition of valinomycin (Fig. 4D). Compared to Wt LmrP, the R260A mutation therefore induces a shift to ΔpH dependence over Δψ dependence in ethidium efflux by the mutant. The energetics of ethidium efflux by the K357R mutant was similar to Wt LmrP, whereas the energetics of the R260K mutant was similar to that of the R260A mutant (data not shown).

### Mutations Affect Hoechst 33342 Transport

**Figure 5:**
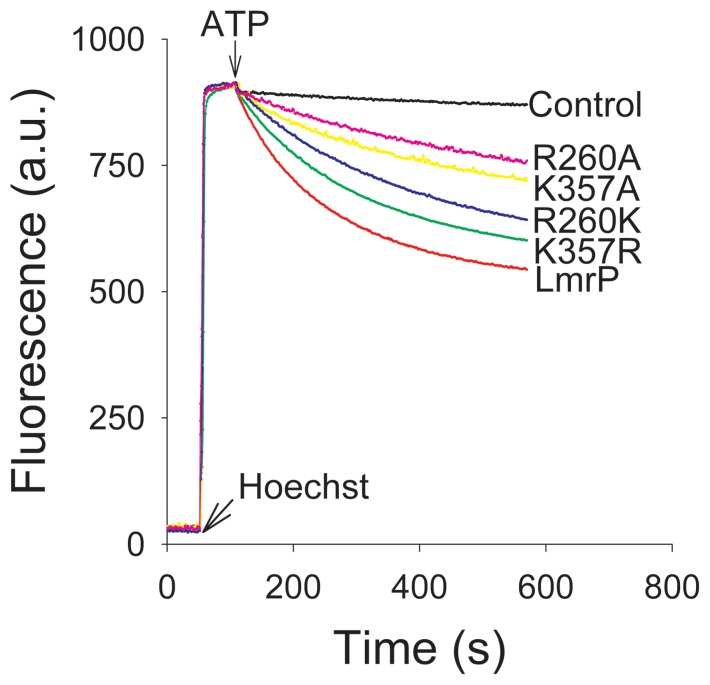
Hoechst 33342 transport in ISOVs containing Wt LmrP, LmrP mutants or no LmrP (control). ISOVs were diluted to a concentration of 0.2 mg protein/ml in 100 mM KP_i_ (pH 7.0) containing 5 mM MgSO_4_, 0.1 mg/ml creatine kinase and 5 mM phosphocreatine. After 50 s, Hoechst 33342 was added to a final concentration of 0.25 µM, followed by the addition of 2 mM ATP at 150 s. Hoechst transport was measured by fluorimetry. Traces represent observations in three independent experiments (n = 3).

Substrate transport by the LmrP mutants was also studied in ISOVs. Hoechst 33342 is a substrate of LmrP [28] and many other MDR transporters [29], [30]. In aqueous solution Hoechst 3342 is essentially non-fluorescent, whereas its partitioning in the membrane is associated with a remarkable enhancement of fluorescence [31]. The fluorescence change of this reaction is reduced at acidic pH, which is most likely due to inhibitory effects of protonation of Hoechst 33342 on the fluorescence quantum yield and on membrane partitioning. In our experiment in ISOVs, a rapid fluorescence intensity enhancement was observed following the addition of 0.25 µM Hoechst 33342 (Fig. 5) due to the partitioning of the dye in the membrane. The addition of ATP to the ISOVs leads to the generation of a Δp (inside acidic and positive) through inward proton pumping by the F_1_F_O_-H^+^ ATPase, which can be used by LmrP proteins to drive proton/Hoechst 33342 antiport. Upon the addition of ATP, ISOVs containing Wt LmrP showed a rapid Hoechst 33342 fluorescence quenching compared to control (Fig. 5) due LmrP-mediated transport of dye from the membrane into the lumen of the ISOVs. The activity of the K357R mutant was closest to that of Wt LmrP. Although reduced, the transport activities of the K357A, R260A and R260K mutants remained substantial (Fig. 5). These findings were consistent with those in ethidium transport measurements (Fig. 3). Further experiments focussed on Wt LmrP and the K357A and R260A mutants.

### Kinetics of Ethidium Transport by LmrP and Mutants

**Figure 6:**
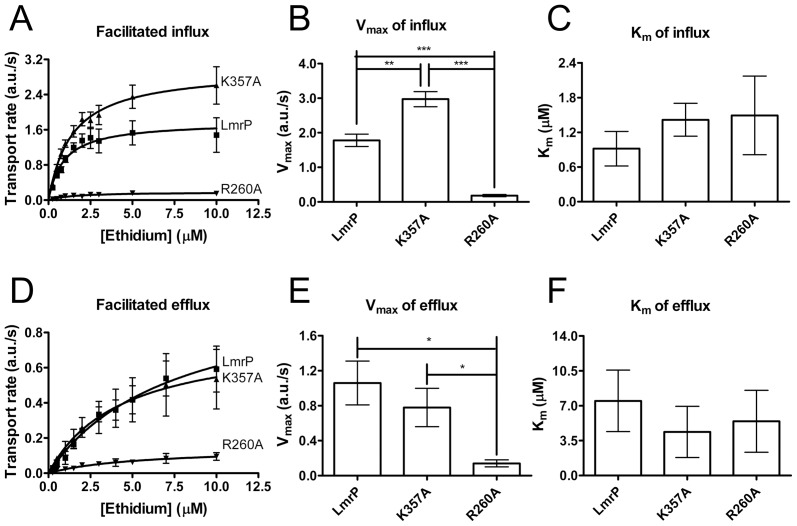
Kinetic analysis of LmrP-mediated, facilitated ethidium influx and efflux. **A**, The ethidium influx rates in ATP-depleted cells were measured as a function of the ethidium concentration. Each data point represents the mean ± SEM of four independent experiments (n = 4). **B,** Comparison of V_max_ of the K357A or R260A mutant with that of Wt LmrP. The data were analysed by one-way ANOVA followed by the post-hoc Tukey’s test. **P<0.01, ***P<0.001, both significantly different. **C,** Comparison of K_m_ for ethidium of the K357A or R260A mutant with that of Wt LmrP. Analysis by one-way ANOVA shows that K_m_ values are not significantly different. **D,** The efflux rates in ATP-depleted cells were measured as a function of the ethidium concentration. Each data point represents the mean ± SEM of three independent experiments (n = 3). **E,** Comparison of V_max_ of K357A or R260A mutant with that of Wt LmrP. The data were analysed by one-way ANOVA followed by the post-hoc Tukey’s test. *P<0.05, significantly different. **F,** Comparison of K_m_ of the K357A or R260A mutant with that of Wt LmrP. K_m_ values were not significantly different when the data were analysed by one-way ANOVA.

Using established methods for LmrA [32], [33] and MsbA [34], the kinetic parameters of the facilitated ethidium influx reaction mediated the LmrP proteins were determined in ATP-depleted cells using ethidium concentrations between 0.25 µM and 10 µM (Fig. 6A). Statistical analysis of the kinetic data revealed a significant increase in the V_max_ of influx for the K357A mutant compared to Wt LmrP (2.97±0.22 a.u./s for K357A mutant vs. 1.78±0.18 a.u./s for Wt LmrP) (Fig. 6B) without a significant change in the apparent K_m_ for ethidium (1.42±0.29 µM for K357A mutant vs. 0.92±0.30 µM for Wt LmrP) (Fig. 6C). The R260A mutation significantly decreased the V_max_ of influx (0.18±0.03 a.u./s for R260A mutant vs. 1.78±0.18 a.u./s for Wt LmrP) (Fig. 6B), whereas no significant change was observed for the apparent K_m_ for ethidium (1.49±0.68 µM for R260A mutant vs. 0.92±0.30 µM for Wt LmrP) (Fig. 6C).

Facilitated efflux of ethidium in cells was measured by imposing an outwardly directed ethidium concentration gradient in the absence of a Δp. ATP-depleted cells in 2 ml of 50 mM KP_i_ buffer (pH 7.0) containing 5 mM MgSO_4_ (A_660_ of 0.5) were equilibrated with ethidium at concentrations ranging from 0.25 µM to 10 µM for 1 h at 30°C in the final volume of 400 µl of the KP_i_ buffer. After equilibration, cells were pelleted by centrifugation. The assay was started by resuspension of the cell pellet in 2 ml of the same buffer without ethidium, and fluorescence was followed over time. The kinetic analysis (Fig. 6D) showed that the V_max_ of facilitated ethidium efflux mediated by K357A mutant is comparable with that of Wt LmrP (0.78±0.22 a.u./s for K357A mutant vs. 1.06±0.25 a.u./s for Wt LmrP) (Fig. 6E). In addition, the apparent K_m_ for ethidium was not significantly affected by the K357A mutation (4.37±2.57 µM for K357A mutant vs. 7.49±3.09 µM for Wt LmrP) (Fig. 6F). However, the R260A mutation significantly decreased the V_max_ of efflux compared to Wt LmrP (0.14±0.04 a.u./s for R260A mutant vs. 1.06±0.25 a.u./s for Wt LmrP) (Fig. 6E), whereas the apparent K_m_ for ethidium was unaltered (5.44±3.11 µM for R260A mutant vs. 7.49±3.09 µM for Wt LmrP) (Fig. 6F). The lack of a significant change in K_m_ for ethidium in the efflux reaction by the LmrP mutants compared to Wt is consistent with the influx data (see above), and suggests that R260 and K357 are not critical for ethidium binding by LmrP.

### Proton Transport

**Figure 7:**
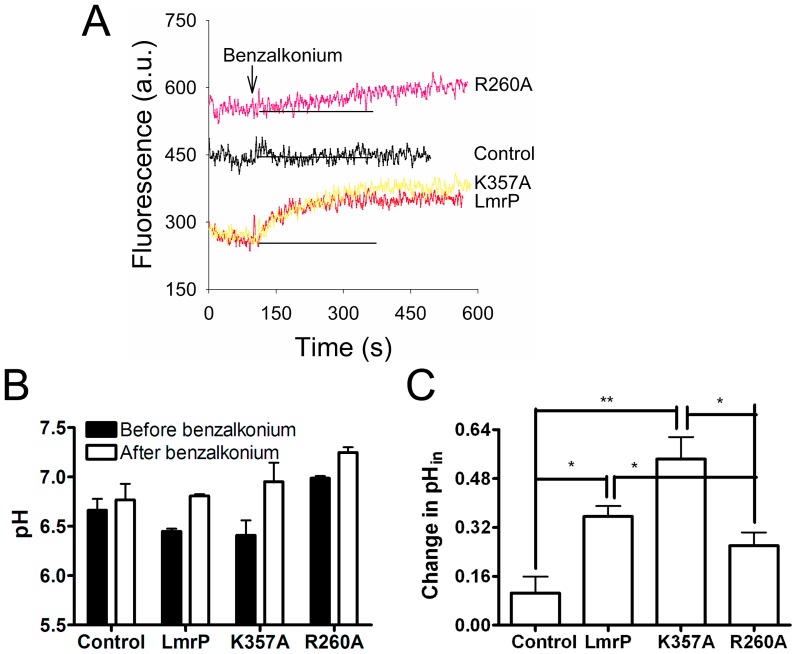
Determination of change in CFSE fluorescence and pH_in_ in intact cells during facilitated benzalkonium influx. **A,** ATP-depleted cells containing Wt LmrP, LmrP mutants (R260A or K357A) or no LmrP protein (Control) were preloaded with 30 µM CFDASE ester, which is intracellulary covalently bound as CFSE, and diluted to a final A_660_ value of 0.5 in 2 ml of 50 mM KPi/5 mM MgSO_4_ at pH 7.0. After the CFSE fluorescence measurement was started, 44.1 µM benzalkonium chloride was added at the time indicated by the arrow. Base line in grey is added to facilitate comparison of the initial slopes (see main text). **B,** The pH_in_ values in cells before and after the addition of the benzalkonium were derived from fluorescence calibration curves (not shown). All values represent the mean ± SEM of three independent experiments (n = 3). **C,** The changes in pH_in_ were analysed using the one-way ANOVA followed by the post-hoc Tukey’s test. *P<0.05, **P<0.01, significantly different. All values represent the mean ± SEM of three independent experiments (n = 3).

The potential role of R260 and K357 in LmrP-mediated proton translocation was also assessed by measuring the change in the intracellular pH (pH_in_) during facilitated influx of substrate in ATP-depleted cells loaded with the fluorescent, covalently-bound and non-transported pH-sensitive probe 5 (and 6-)-carboxyfluorescein succinimidyl ester (CFSE). Although ethidium is often used as a test substrate for LmrP, its excitation and emission spectra overlap with those of CFSE. Therefore, the non-fluorescent cation benzalkonium^+^ was used as an alternative substrate. During facilitated influx of benzalkonium by Wt LmrP in ATP-depleted cells at an external concentration of 44.1 µM, the fluorescence of the pH probe increased compared to the non-expressing control (Fig. 7A), pointing to an increase in the intracellular pH (pH_in_) during proton/benzalkonium antiport. The addition of the benzalkonium also resulted in an increased fluorescence of CFSE in cells expressing the K357A or R260A mutants compared to control (Fig. 7A). Calculation of pH_in_ values from the linear fluorescence increase during the first 40 s of facilitated benzalkonium influx by Wt LmrP and K357A LmrP, and the first 150 s for R260A LmrP and Control, show that the pH_in_ increased from 6.45±0.03 to 6.81±0.02 for Wt LmrP, 6.41±0.15 to 6.95±0.19 for mutant K357A, and 6.99±0.02 to 7.25±0.05 for mutant R260A, whereas the pH_in_ in the control cells remained relatively constant between 6.66±0.11 to 6.77±0.16 during the experiment (Fig. 7B). Hence, for all LmrP proteins, the pH_in_ was significantly elevated compared to the pH_in_ in control cells (Wt LmrP and R260A LmrP vs. Control, P<0.05; K357A LmrP vs. Control, P<0.01; K357A LmrP vs. R260A LmrP, P<0.05). The change in pH_in_ in cells was largest for Wt LmrP and the K357A mutant, and smallest for the R260A mutant (Fig. 7C), consistent with the effect of these mutations on the V_max_ of substrate transport (Fig. 6E). Taken together, the data that K357 and R260 are not critical for proton translocation.

### Conformational Changes

**Figure 8:**
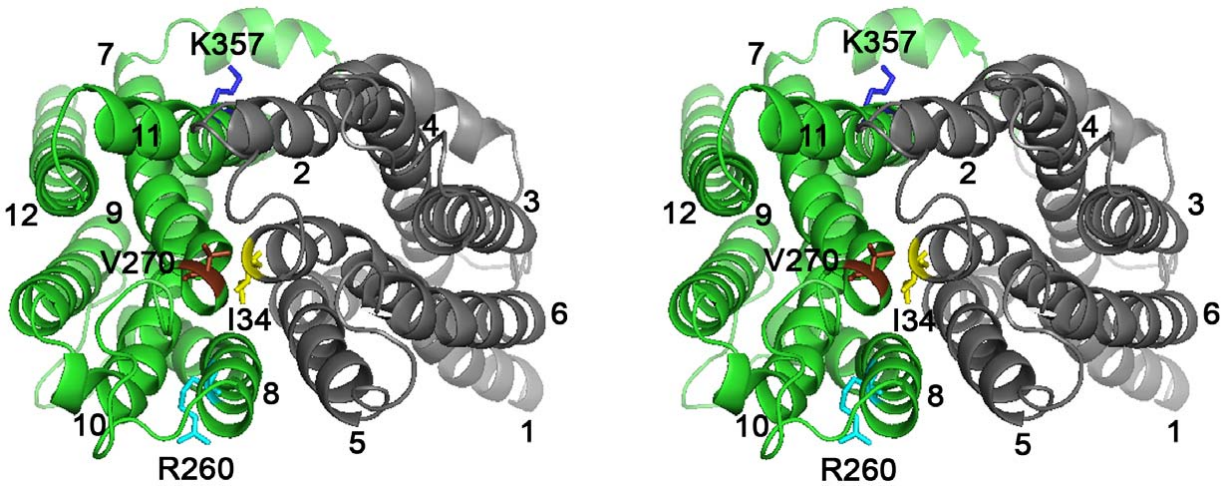
Close proximity between I34 and V240 in inward-facing LmrP. Stereoview of inward-facing model [12] viewed from external membrane surface, showing I34 (TMH 1) in yellow and V240 (TMH 7) in brown, and the basic residues R260 (TMH 8) in light blue and K357 (TMH 11) in dark blue. I34 and V240 were substituted by C in cysteine-less LmrP (referred to LmrP-*cl*), yielding the double cysteine mutant I34C V240C LmrP-*cl* (referred to as LmrP-*dc*).

**Figure 9:**
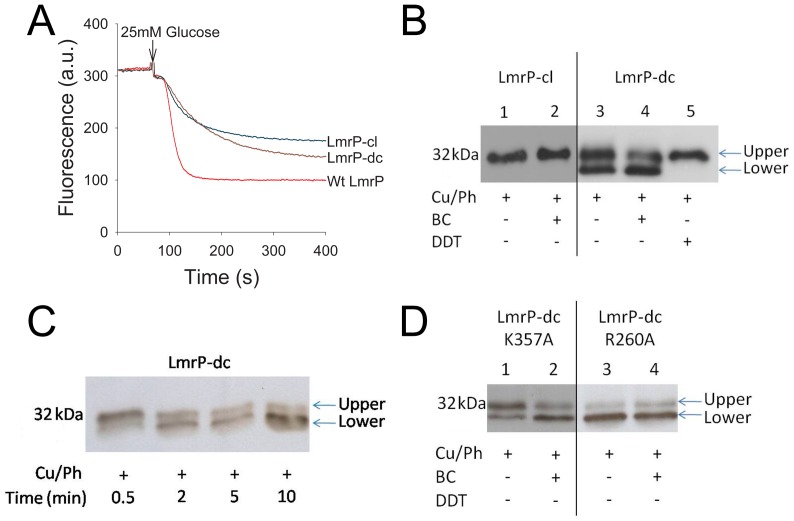
Intramolecular disulfide formation in LmrP*-dc* (I34C & V240C) and mutants derived thereof. **A**, Ethidium efflux in intact cells expressing Wt LmrP, LmrP*-cl* or LmrP*-dc* show that, similar to Wt LmrP, the mutants are transport active. The measurements were performed as described in the legend to Fig. 3A. The traces represent results of three independent experiments (n = 3). **B,** Cysteine crosslinking between I34C and V240C in LmrP*-dc.* RSOVs containing LmrP*-dc* (2 µg protein/µl) in 100 mM KP_i_ buffer, pH 7.0, plus 5 mM MgSO_4_ were exposed to 0.5 mM copper phenanthroline (*Cu/Ph*) in the presence or absence of 97.9 µM benzalkonium chloride (*BC*) for 5 min at 10°C. The reactions were stopped by the addition of excess (10 mM) of the thiol alkylator N-ethylmaleimide followed by incubation for 2 min at 10°C. Total protein (5 µg) of RSOVs from the crosslinking reactions were mixed with 5 × SDS sample-loading buffer devoid of dithiothreitol (DTT) (*lane 1–4*) or containing 2 mM DTT (*lane 5*), and separated on 11% SDS-PAGE. The SDS-PAGE gel was subsequently subjected to Western blot transfer onto a HybondP membrane. Transferred proteins were probed with anti-His_5_ antibody. Upper band: uncrosslinked protein; Lower band: crosslinked protein. **C,** Time course for disulfide formation between I34C and V240C. The crosslinking in RSOVs containing LmrP*-dc* was followed for 10 min at 10°C as described for panel B, lane 3. Samples of the crosslinking reactions (2 µg) were mixed with 5 × SDS sample-loading buffer devoid of DTT, and separated on SDS-PAGE. **D,** The effect of K357A or R260A mutation on disulfide formation between I34C and V240C. The crosslinking was performed as described in panel B for 5 min at 10°C. Equal loading of lanes was confirmed by densitometry of the same samples on Coomassie-stained SDS-PAGE (not shown).

To investigate whether the changes in the V_max_ of substrate transport by the K357A and R260A LmrP mutants are related to changes in conformational transitions between inward-facing and outward-facing states, the conformational changes in the K357A and R260A mutants were compared with LmrP itself in a Cys-less background (LmrP*-cl*) in which cysteine residues were inserted at position I34 and V240 (double cysteine mutant LmrP*-cl* I34C/V240C, referred to as LmrP*-dc*) (Fig. 8). Recently, an analogous strategy was incorporated in studies on conformational changes in the MFS transporter LacY [35], [36]. According to our structure model of LmrP, the Cys side chains at positions I34 and V240 would be close enough to form a disulfide crosslink in the inward-facing conformation (Fig. 8) but not in the outward-facing conformation where, based on analogous outward-facing models for secondary-active membrane transporters such as LacY, LeuT and Mhp1 [15], [16], [37], these residues would be separated by at least 20 Å. In an ethidium transport assay in which cells were preloaded with 2 µM ethidium, the LmrP*-cl* mutant and the LmrP*-dc* mutant retained significant active ethidium efflux activity compared to Wt LmrP (2.22±0.05 a.u./s, 1.38±0.05 a.u./s, and 5.63±0.40 a.u./s, respectively, P<0.001) (Fig. 9A). Hence, the LmrP*-dc* mutant can be used to study conformational changes associated with substrate transport.

When total membrane protein from RSOVs was incubated under oxidizing conditions by exposure to copper phenanthroline, and subsequently separated on SDS-PAGE gel and analysed by Westernblot probed with anti His-tag antibody, LmrP*-cl* protein was detected with a single signal corresponding to a molecular mass of about 32 kDa (Fig. 9B, *lane 1*). In contrast, if this experiment was repeated with LmrP*-dc*, this double cysteine mutant gave rise to two signals. Whereas the upper signal corresponds to the same molecular mass as LmrP*-cl* and represents uncrosslinked protein, the lower signals runs at a smaller apparent molecular mass (Fig. 9B, *lane 3*). Interestingly, when followed over an extended incubation period of 10 min, the intensity of the lower signal progressively increased whereas the intensity of the upper signal reduced during this time (Fig. 9C). The notion from these experiments that this lower signal was associated with intramolecular disulfide crosslinking in LmrP*-dc* was confirmed by the observation that the lower band was resolved into the upper band when the protein samples were diluted in loading buffer containing dithiothreitol (DTT), prior to separation on the SDS-PAGE gel (Fig. 9B, *lane 5*). We anticipate that the intramolecular crosslinking in LmrP results in its incomplete unfolding in denaturing SDS-PAGE, causing a shift to a lower apparent molecular mass.

**Figure 10:**
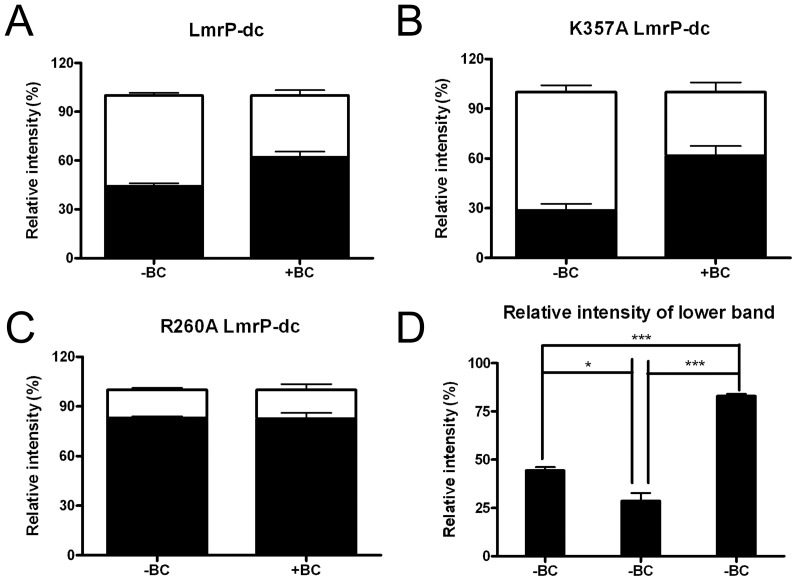
Densitometric analyses of disulfide formation by LmrP*-dc*, K357A LmrP*-dc* and R260A LmrP*-dc*. The white and black bars represent the relative signal intensity of the upper and lower bands, respectively, on the Westernblots in panels B–D in Fig. 9, relative to the sum of upper and lower bands signals (with an intensity of 100%). A, LmrP-*dc*, B, K357A LmrP-*dc*, C, R260A LmrP-*dc*, in the absence or presence of benzalkonium chloride. D, Relative signal intensity of the lower band (reflecting inward-facing conformation) for LmrP-*dc*, K357A LmrP-*dc* and R260A LmrP-*dc*, respectively, in the absence of benzalkonium chloride. Error bars represent ± SEM and are based on three independent experiments. Unpaired student t tests were used for panels A, B, and C, to compare the density of upper and lower signals under the same treatment, whereas paired student t-tests were used to compare the density of the upper band signal or lower band signal in the absence and presence of benzalkonium. One-way ANOVA and the post-hoc Tukey’s test were performed for the analyses in panel D, to determine whether the density of the lower bands of the LmrP mutants were significantly different from each other. *P<0.05, ***P<0.001, significantly different. Upper band: uncrosslinked protein; Lower band: crosslinked protein.

The presence of the transported substrates ethidium or benzalkonium stimulated the disulfide crosslinking reaction in LmrP*-dc*. Unpaired student t test for the density of upper and lower band signals in Fig. 9B (*lane 3*) after crosslinking for 5 min in the absence of substrate indicated that less than half of the population of LmrP*-dc* was in the crosslinked state (crosslinked LmrP*-dc* versus uncrosslinked LmrP*-dc*: 44.4±1.8% vs. 55.6±1.8%, P<0.05) (Fig. 10A). In the presence of 97.9 µM benzalkonium (Fig. 9B, *lane 4*), a substantially larger population of LmrP*-dc* was found in the crosslinked conformation (crosslinked LmrP*-dc* vs. uncrosslinked LmrP*-dc*: 62.2±3.4% vs. 37.8±3.4%, P<0.01) (Fig. 10A). In a complementary comparison, paired student t tests for density of the lower band signal in the absence and presence of benzalkonium indicated that the population of crosslinked LmrP*-dc* significantly increased (44.4±1.8% vs. 62.2±3.4%, P<0.05) (Fig. 10A), whereas paired student t test for densitometry of upper band signal in the absence and presence of benzalkonium suggested that the population of uncrosslinked LmrP*-dc* significantly decreased (55.6±1.8% vs. 37.8±3.4%, P<0.05) (Fig. 10A). In contrast to these findings for LmrP*-dc*, the exposure of LmrP*-cl* to benzalkonium did not have any effect on the mobility or intensity of its signal (Fig. 9B, *lane 2*). Similar observations were made with ethidium (data not shown), but the band shifts were stronger with benzalkonium.

Consequently, the data show that at the end of the 5-min period of crosslinking, the dominant population of LmrP*-dc* in the RSOVs is in an outward-facing conformation. Upon the addition of a transport substrate, the two six-helix bundles formed by the N- and C-terminal halves are drawn closer together at the external side of the protein due to high-affinity binding of this substrate to the inward-facing state, thus increasing the population of LmrP*-dc* in the inward-facing conformation. In the inward-facing state, the I34C and V240C side chains are close enough to form an intramolecular disulfide bond.

### Effect of K357A and R260A Mutations on Crosslinking

The cysteine crosslinking assay was used to examine the conformational transitions of the K357A and R260A LmrP*-dc* mutants and the effect of benzalkonium on these transitions. For the experimental data for K357A LmrP*-dc* in the absence of benzalkonium (Fig. 9D, *lane 1*), the unpaired student t-test for densitometry of signals indicate that at the end of the 5 min period of crosslinking, the population of crosslinked protein was smaller than the population of the uncrosslinked protein (28.6±4.1% vs. 71.4±4.1%, P<0.01) (Fig. 10B). The presence of benzalkonium (Fig. 9D, *lane 2*) significantly increased the population of crosslinked K357A LmrP*-dc* compared to its absence (61.7±5.9% vs. 28.6±4.1%, P<0.05), whereas the population of uncrosslinked LmrP significantly decreased (71.4±4.1% vs. 38.3±5.9%, P<0.05) (Fig. 10B). Hence, as observed for LmrP*-dc*, benzalkonium binding to LmrP*-dc* K357A in RSOVs shifts the population of LmrP from outward-facing to inward-facing.

For the LmrP*-dc* R260A mutant in the absence of benzalkonium, the population of crosslinked protein was much larger than that of uncrosslinked protein (82.9±1.3% vs. 17.1±1.1%, P<0.001) (Fig. 9D, *lane* 3; Fig. 10C, D). Strikingly, in the presence of benzalkonium, this difference remained largely unaltered (82.9±1.1% vs. 17.4±3.6%) (Fig. 9D, *lane* 4; Fig. 10C, D). Meanwhile, paired student’s t-test for the density for the uncrosslinked protein in the absence and presence of benzalkonium remained almost the same (17.1±1.1% vs. 17.4±3.6%) (Fig. 10C). These observations show that by the end of the crosslinking reaction with LmrP*-dc* R260A, the predominant population in the RSOVs is in an inward-facing conformation, and that this distribution is unaffected by the addition of benzalkonium (Fig. 10C). These findings contrast with those on K357A LmrP*-dc*, which in the absence of added benzalkonium formed a significantly smaller population of crosslinked protein compared to LmrP*-dc* itself (28.6±4.1% vs. 44.4±1.8%, P<0.05) (Fig. 10D), suggesting a reduced population of K357A LmrP-*dc* in the inward-facing conformation. Hence, the data indicate that substitution of K357 and R260 to A shifts roughly equal populations of inward-facing and outward-facing LmrP in the crosslinking reaction towards enlarged populations of outward-facing LmrP (K357A) and inward-facing LmrP (R260A), respectively.

## Discussion

In our transport experiments, K357A and R260A mutations did not affect the K_m_ for ethidium (Fig. 6). However, the R260A mutant showed a substantial reduction in the V_max_ compared to Wt LmrP and the K357A mutant (Fig. 6). This difference in V_max_ was also reflected in the proton transport activities during benzalkonium-proton exchange, which were low for the R260A mutant compared to Wt LmrP and the K357A mutant (Fig. 7). The findings indicate that while K357A and R260A mutations affect ethidium-proton and benzalkonium-proton antiport, neither K357 nor R260 is critical in substrate binding or proton translocation. This conclusion might relate to the difference in the location of K357 and R260 in our inward-facing LmrP model compared to the location of the catalytic carboxylates D142, D235 and E327 with established roles in substrate binding and/or proton coupling [22]. Whereas the catalytic carboxylates co-localise on the surface of the interior cavity [12], K357 and R260 are present on the outside surface of LmrP, where they are exposed to the phospholipid bilayer.

The intra-molecular cysteine crosslinking experiments with LmrP-*dc* (Fig. 9) are in agreement with the current view that members of the MFS can be present in at least two major conformational states that are interconverted by the rigid-body movement of both halves of the transporter, i.e., the inward-facing conformation in which I34C and V240C in LmrP can interact with each other, and the outward-facing conformation in which these cysteines are too far apart for cross-linking (Fig. 8). Together with the correct predictions by our LmrP model on the catalytic role of D142, D235 and E327 in proton and substrate interactions [12], [22], the observed cross-linking between the two cysteines in LmrP-*dc* (Fig. 8) further supports the accuracy of the model (Fig. 8) [12]. The substitution of K357 and R260 by A changed the distribution of LmrP-*dc* over these protein conformations during the crosslinking reaction (Figs. 9,10). At the end of the reaction, the population of K357A LmrP*-dc* in the outward-facing state was larger than for LmrP*-dc*, but the transition to the inward-facing state could still be established by the addition of benzalkonium (Fig. 10). Under identical conditions, R260A LmrP*-dc* was mostly present in the inward-facing conformation, and the presence of benzalkonium did not induce conformational transitions (Figs. 9,10).

As K357 and R260 are located close to the head group region of the phospholipid bilayer, they might affect the conformations of LmrP through direct interactions with phospholipids in the interfacial region. In X-ray crystal structures of various membrane proteins, such as the yeast cytochrome c oxidase [38] and cytochrome *bc*_1_ complex [39], protein-lipid interactions have been observed that are based on electrostatic interactions between the phosphodiester layer of the membrane and a basic amino acid side chain. In crystal structures of the mammalian voltage-sensitive K^+^ channels KvAP [40], [41] and Kv1.2 [42], and the lipid- and mechano-sensitive K^+^ channel K2P TRAAK [43], this type of interaction was found to be critical for the stability of conformational states that occur during gating. Our finding that the Δp-dependent ethidium and Hoechst 33342 transport activity of mutants with conservative R260K and K357R replacements was closer to Wt LmrP than non-expressing control, whereas the activity of the R260A and K357A mutants was closer to the non-expressing control than Wt LmrP (Figs. 3,5) points to an important functional role of a basic side chain at these positions. It is noteworthy that R260 is highly conserved in orthologs of LmrP in species including *Streptococcus*, *Enterococcus*, *Staphylococcus*, *Macrococcus*, *Bacillus*, *Weissella*, *Leuconostoc* and others. Amongst these orthologs, the conservation of K357 appears to be more limited to those in *Weissella* and *Leuconostoc* species.

**Figure 11:**
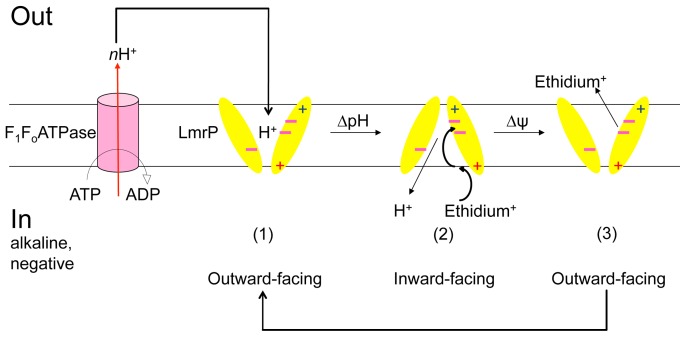
Alternating access model for Δp-dependent ethidium efflux by LmrP. Proton extrusion by the F_1_F_O_-H^+^ ATPase (in purple) in cells generates a Δp (interior negative and alkaline) across the cytoplasmic membrane. The N- and C-terminal halves of LmrP are represented by yellow ellipses. Minus signs in pink represent the catalytic carboxylates (D142, D235 and E327) that are involved in substrate/proton interactions. During electrogenic efflux via proton-ethidium antiport, carboxylates are protonated in the outward-facing conformation (1), which triggers a ΔpH-dependent conformational switch to the inward-facing conformation (2). Drug binding and proton release at the inside surface will facilitate a second conformational switch in which dissociated carboxylates reorient back to the outward-facing conformation (3) in a Δψ-dependent fashion. Conformation (3) might be the same as conformation (1). K357 (red plus sign, TMH 11) and R260 (blue plus sign, TMH 8) are located on the outer surface of LmrP, near the phospholipid head group region of the inner leaflet (K357) and outer leaflet (R260) of the membrane. In our experiments, alanine replacements at position 357 and 260 shift roughly equal populations of inward-facing and outward-facing LmrP towards outward-facing (K357A) and inward-facing (R260A) populations.

The notion that the K357 and R260 influence transitions between the inward-facing state and outward-facing state of LmrP could explain the energetics of active ethidium efflux by the K357A and R260A mutants (Fig. 4). Although it is known that LmrP can utilise the Δp (interior negative and alkaline) to drive ethidium efflux from cells, it is not established in detail how Δψ and ΔpH dependence are related to the conformational changes during the drug transport cycle. If the carboxylates responsible for proton coupling (D142 in TMH5, D235 in TMH7, and E327 in TMH10 [22]) are protonated when exposed to the outside surface of the membrane and deprotonated in the inward-facing state, the transition from the outward-facing state to the inward-facing state would be ΔpH dependent. Our recent experiments imply that this is indeed the case [22]. By analogy to voltage-gated K^+^ channels [42], basic residues in TMHs in LmrP could impose Δψ dependence when they would move in a protonated (cationic) state from the electrical outside to the electrical inside. However, LmrP does not contain basic residues in central regions of the TMHs that form reorienting surfaces, and it is therefore more likely that Δψ dependence in LmrP originates from the reorientation of the dissociated (anionic) carboxylates during the transition from the inward-facing state to the outward-facing state (Fig. 11). Our previous observations on LmrP that any single carboxyl to neutral amide replacement at positions D142, D235 or E327 changes electrogenic 3H^+^-propidium^2+^ antiport to electroneutral 2H^+^-propidium^2+^ antiport [22], is consistent with this proposal. Hence, the Δψ and ΔpH might act on different parts of the transport cycle (the transition from 2 to 3, and the transition from 1 to 2, respectively). The finding that the K357A mutation diminishes the population of LmrP in the inward-facing conformation (state 2 in Fig. 11) might hamper the Δψ-dependent progression of the transport cycle from the inward-facing state to the outward-facing state (state 3 in Fig. 11), which could offer an explanation for the bias towards Δψ dependence of ethidium efflux by this mutant (Fig. 4A). Likewise, as the R260A mutation reduces the population of LmrP in the outward-facing conformation (state 1 in Fig. 11), this phenotype might hinder the ΔpH-dependent progression of transport cycle from the outward-facing state to the inward-facing state, and might thus increase the ΔpH dependence during ethidium efflux (Fig. 4B). It can be anticipated that the replacement of residues in loop regions that connect TMHs might also affect the distribution of transport proteins over conformational states, when these residues support the stability of these states through loop-loop and/or loop-helix interactions. Past conclusions on the direct role of D68 and D128 in the intracellular loop between TMH 2 and 3, and TMH 4 and 5, respectively, in proton-coupled transport by LmrP ([44], [45]) might therefore require further investigations.

In conclusion, our data on LmrP suggest that K357 and R260 are not directly involved in interactions with transported substrates or coupling protons. Instead, these residues affect the dynamics of major protein conformations in the transport cycle, potentially through their interactions with phospholipids and/or other residues in LmrP. The replacement of these residues by alanine therefore changes the V_max_ at which the transport cycle can operate. The K357A and R260A substitutions also influence the energetics of transport, which might relate to the notion that transitions between conformational states are differently affected by components of the proton-motive force.

## Materials and Methods

### Construction of Mutants

The mutations K357R, K357A, R260K, or R260A were generated in C-terminally His_6_-tagged LmrP in the *E. coli* pGEM-5Zf(+) (Promega) derivative pGHLmrP [12] by site-directed mutagenesis using the QuikChange kit (Stratagene) with forward and reverse primers as listed in Table 1. Using the same method, the endogenous C270 in LmrP was replaced by S to create the cysteine-less protein (LmrP*-cl*) (Table 1). The PCR product was digested with NcoI and BamHI, and cloned into pGH_10_ABCG2 (an *E. coli vector* encoding C-terminally His_10_-tagged ABCG2) in exchange for the *ABCG2* gene, yielding pGH_10_LmrP*-cl*. The double-Cys I34C/V240C mutant of LmrP (LmrP*-dc*) was generated in pGH_10_LmrP*-cl* using the primers listed in Table 1. Finally, the K357A and R260A mutants were generated in pGH_10_LmrP*-dc* using the primers listed in Table 1. The cloned PCR products were sequenced to ensure that only the intended changes were introduced. The mutant *lmrP* genes were then subcloned as NcoI-XbaI fragments into pNZ8048 [24] downstream of the nisin A-inducible promoter, yielding pNZLmrP K357R, pNZLmrP K357A, pNZLmrP R260K, pNZLmrP R260A, pNZLmP*-cl*, pNZLmrP*-dc*, pNZLmrP*-dc* K357R, pNZLmrP*-dc* K357A, pNZLmrP*-dc* R260K, and pNZLmrP*-dc* R260A.

### Bacterial Strains, Plasmids and Growth Conditions

*L. lactis* strain NZ9000 Δ*lmrA* Δ*lmrCD* [23], which is devoid of the endogenous multidrug transporters LmrA and LmrCD, was used as a host for pNZ8048-based plasmids [24]. Cells were grown in M17 broth (Oxoid Ltd., Basingstoke, UK) supplemented with 0.5% glucose at 30°C. Where required, chloramphenicol was added to a final concentration of 5 µg/ml. Medium was inoculated with a 1∶50 dilution of an overnight cell culture, and cells were grown to an A_660_ of 0.5–0.6. Expression of proteins was then induced for 1 h at 30°C in the presence of a 1∶1000 dilution of the supernatant of an overnight culture of the nisin-producing strain *L. lactis* NZ9700 by previously described methods [12]. *E. coli* strain XL1 Blue was used as a host for pGHLmrP and derivatives, and was grown at 37°C under aerobic conditions on an orbital shaker in Luria-Bertani (LB) Broth (Difco) containing 50 µg/ml carbenicillin where required.

### Preparation of Inside-out Membrane Vesicles (ISOVs)

ISOVs were prepared as described previously [22], suspended in 100 mM KP_i_ (pH 7.0) containing 10% glycerol, and stored in liquid nitrogen. The protein concentration was determined using the Bio-Rad DC assay (BioRad, Richmond, CA, USA), and expression of the His-tagged proteins was confirmed on Western blot probed with anti-His_5_-tag antibody (Sigma-Aldrich, St. Louis, MO, USA).

### Preparation of Right-side-out Membrane Vesicles (RSOVs)

RSOVs were prepared by osmotic lysis as described previously [46], suspended in 50 mM KPi buffer (pH 7.0) containing 10% glycerol at a protein concentration of about 5–10 mg/ml, frozen in liquid nitrogen and stored at −80°C until use. The protein concentration was determined using the Micro BCA Protein Assay (Thermo Scientific Pierce, Rockford, IL, USA), and expression of the His-tagged proteins was confirmed on Western blot probed with anti-His_5_-tag antibody (Sigma-Aldrich, St. Louis, MO, USA).

### Immunoblotting

Total membrane protein in ISOVs and RSOVs were subjected to 10% SDS-PAGE. Proteins were then electroblotted to Hybond-P membrane (Immobilon Transfer Membrane; Milipore Corp., Billerica, MA, USA) and were detected in the presence of a 1∶1000 dilution of the monoclonal anti-His_5_-tag antibody (Sigma Aldrich). The primary antibody was probed with a horseradish peroxidise (HRP)-coupled secondary anti-mouse antibody (Sigma-Aldrich). Detection of the secondary antibody was performed using the enhanced chemiluminescence system (ECL; Pierce, Rockford, IL, USA).

### Protease Digestion of C-terminal Histidine-tag

To compare the accessibility to protease digestion of the intracellular His-tag of Wt LmrP versus mutant LmrP proteins in ISOVs and RSOVs, 95 µg total membrane protein in 47.5 µl of 50 mM (K)HEPES buffer (pH 7.5) supplemented with 1 mM CaCl_2_, was incubated in the presence of 3.8 µg proteinase K [27]. After incubation at 37°C, the reaction was terminated at the times indicated in Fig. 2B by the addition of 2.5 µl of 200 mM phenylmethanesulphonyl fluoride (PMSF, in ethanol), and then mixed with 10.4 µl of 6x SDS-loading buffer. Total membrane protein (15 µg) of each sample was subjected to SDS-PAGE followed by immunoblotting using the monoclonal anti-His_5_-tag antibody.

### Ethidium Transport in Intact Cells

Cells were grown to an A_660_ of 0.5–0.6 from overnight cultures in fresh M17 medium at 30°C. The cells were harvested by centrifugation at 6,500 g for 8 min at 4°C and washed once with 50 mM KP_i_ buffer (pH 7.0) containing 5 mM MgSO_4_. Cells were resuspended to an A_660_ of 1.2 in the same KP_i_ buffer containing 0.5 mM of the uncoupler 2,4-dinitrophenol, and then incubated for 45 min at 30°C [32]. The cells were subsequently washed three times with ice-cold 50 mM KP_i_ buffer containing 5 mM MgSO_4_ at pH 7.0, and resuspended in this buffer to an A_660_ of 5.0 and kept on ice.

To measure facilitated ethidium influx, ATP-depleted cells were diluted 10-fold in 2 ml of 50 mM KP_i_ buffer containing 5 mM MgSO_4_ at pH 7.0. After 80 s, the required concentration of ethidum bromide ranging from 0.25 µM to 10 µM was added to the cells, and fluorescence was followed over time in a Perkin Elmer LS 55B fluorimeter using excitation and emission wavelengths of 500 and 580 nm, respectively, and slit widths of 5 and 10 nm, respectively. The influx rates in Fig. 6A were calculated over the first 25 s of uptake during which uptake was linear. Rates of passive influx by nonexpressing control cells were subtracted from those obtained for cells expressing Wt LmrP or LmrP mutants. To measure facilitated ethidium efflux, ATP-depleted cells in 2 ml of 50 mM KP_i_ buffer containing 5 mM MgSO_4_ at pH 7.0 were pelleted by centrifugation, and loaded with ethidium bromide at required concentrations ranging from 0.25 µM to 10 µM for 1 h at 30°C in a final volume of 400 µl of the same buffer. After loading with ethidium, cells were pelleted at 15,000 g for 2 min. The assay was started by resuspension of the cell pellet in 2 ml of the same buffer without ethidium bromide, and fluorescence was followed over time. The efflux rates in Fig. 6D were calculated over the first 20 s of efflux during which efflux was linear. Rates of passive efflux by nonexpressing control cells were subtracted from those obtained for cells expressing Wt LmrP or LmrP mutants.

Active ethidium efflux was monitored in cells preloaded with 2 µM ethidium bromide [32]. The subsequent addition of 20 mM glucose to the ATP-depleted cells allowed the generation of a Δp (inside alkaline and negative) via H^+^ extrusion by the F_O_F_1_ ATPase. The Δp-dependent LmrP-mediated efflux of ethidium was then monitored by fluorimetry. To manipulate the composition of the Δp, the ionophores nigericin and valinomycin were used at final concentrations of 1 µM each, and were added 3 min before the addition of glucose.

### Hoechst 33342 Transport

Transport of Hoechst 33342 (2′-(4-Ethoxyphenyl)-5-(4-methyl-1-piperazinyl)-2,5′-bi-1H-benzimidazole) in ISOVs was conducted as described previously [47]. ISOVs (400 µg of total protein) were diluted into 2 ml of 100 mM KPi buffer (pH 7.0) containing 5 mM MgSO_4_, 0.1 mg/ml creatine kinase, and 5 mM phosphocreatine (both Roche Diagnostics Ltd, Burgess Hill, UK). After 50 s, Hoechst 33342 (0.25 µM) was added, and the fluorescence was monitored until a steady state was reached. Subsequently, sodium ATP (in 50 mM KPi (pH 7.0)) was added to a final concentration of 2 mM, and the fluorescence intensity was followed further over time. Hoechst 33342 fluorescence was measured at excitation and emission wavelengths of 355 and 457 nm, respectively, and slit widths of 10 and 5 nm, respectively.

### Measurement of Intracellular pH (pH_in_) during Facilitated Benzalkonium Influx

The ATP-depleted cells were prepared as described under “Ethidium transport in intact cells”. Subsequently, the cells were loaded with the fluorescent pH-sensitive probe, 5 (and 6-)-carboxyfluorescein diacetate *N*-succinimidyl ester, CFDASE, as described previously [48]–[50] with modifications. The cells were incubated for 30 min at 30°C in the presence of 30 µM CFDASE, washed, and resuspended in 50 mM KP_i_ buffer (pH 7.0) containing 5 mM MgSO_4_. To eliminate unconjugated CFSE, 10 mM glucose was added, and the cells were incubated for an additional 30 min at 30°C. The cells were then washed twice, resuspended in the 50 mM KP_i_ buffer (pH 7.0) containing 5 mM MgSO_4_, concentrated to a final A_660_ value of 20, and placed on ice until required.

To measure the effect of benzalkonium chloride on the pH_in_, the concentrated CFSE-loaded cell suspension was diluted 40-fold in 2 ml of 50 mM KP_i_ buffer (pH 7.0) containing 5 mM MgSO_4_. Fluorescence was measured at excitation and emission wavelengths of 490 nm 520 nm respectively and slit widths of 5 nm each. After 200 s, 15 µg/ml benzalkonium chloride (corresponding to 44.1 µM) was added. The assay mixture in the cuvette was then immediately used for calibration of the fluorescence. After the Δψ and ΔpH were dissipated in the presence of 1 µM valinomycin plus 1 µM nigericin (pH_in_ = pH_out_), respectively, the pH of the buffer was adjusted with either HCl or NaOH to pH values between 5.5 and 8.0 using a pH micro-electrode, after which CFSE fluorescence was measured.

### Intramolecular Cysteine Crosslinking

For each crosslinking reaction, RSOVs were diluted to 2 µg protein/µl in 100 mM KP_i_ buffer, pH 7.0, containing 5 mM MgSO_4_ in a reaction volume of 30 µl in microcentrifuge tubes with pierced lids. To reduce background signals due to crosslinks formed before benzalkonium chloride addition, 0.5 mM DTT was added, and the samples were incubated for 2 min at 10°C [51]. Benzalkonium chloride was then added to a final concentration of 33.3 µg/ml (corresponding with 97.9 µM), and the crosslinking reactions were initiated by the addition of 0.5 mM copper phenanthroline solution, which was added from a 50 mM stock made by mixing CuSO_4_ and 1,10 phenanthroline in a ratio of 1∶4 (w:w) in ultrapure H_2_O. Some minor precipitates during the crosslinking reactions were re-dissolved by mixing gently, and the reaction was allowed to occur at 10°C at incubation times of 5 min (Fig. 9B, D) or 10 min (Fig. 9C). The reactions were stopped by the addition of excess (10 mM) of the thiol alkylator N-ethylmaleimide and incubation for 2 min at 10°C. To prevent cleavage of formed disulfide crosslinks, 5 µg of protein of RSOVs from the crosslinking reactions was mixed with 5x SDS sample-loading buffer devoid of DTT, and separated on an 11% SDS-PAGE without any incubation. Gels were analysed by Western Blotting using anti-His_5_-tag antibody as described under “Immunoblotting”. Band intensities on Western blot were compared by densitometric analyses using ImageJ software version 1.43 (NIH).

### Statistical Methods

The kinetics of facilitated ethidium influx and efflux was fitted to a hyperbola using the equation: y  =  ax/(b + x), in which y is the transport rate in a.u./s, x is the ethidium concentration in µM, a is the maximum rate of the reaction (V_max_) in a.u./s, and b (Michaelis constant, K_m_) is the ethidium concentration (µM) giving ½V_max_. The calibration curves that provide the relationship between the pH_in_ and the CFSE fluorescence in cells were fitted according to a four parameter sigmoid function as described previously [48]. All experiments were performed at least three times on independent occasions. Significance was calculated using the student t-test and ANOVA. Statistical significance was calculated at a confidence interval of at least 95%.
